# Factors associated with the performance of routine health information system in Yaoundé-Cameroon: a cross-sectional survey

**DOI:** 10.1186/s12911-020-01357-x

**Published:** 2020-12-17

**Authors:** Georges Nguefack-Tsague, Brian Bongwong Tamfon, Ismael Ngnie-Teta, Marie Nicole Ngoufack, Basile Keugoung, Serge Marcial Bataliack, Chanceline Bilounga Ndongo

**Affiliations:** 1 Department of Public Health, Faculty of Medicine and Biomedical Sciences, University of Yaoundé 1, Yaoundé, Cameroon; 2 Challenges Initiative Solutions, Yaoundé, Cameroon; 3 Helen Keller International, Yaoundé, Cameroon; 4 Department of Biochemistry, Faculty of Science, University of Yaoundé 1, Yaoundé, Cameroon; 5 Systems Biology, Chantal Biya International Reference Centre for Research on HIV and AIDS Prevention and Management (CBIRC), Yaoundé, Cameroon; 6 UNICEF, Yaoundé, Cameroon; 7 World Health Organisation, Yaoundé, Cameroon; 8 Department of Disease Control, Epidemics and Pandemics, Ministry of Public Health, Yaoundé, Cameroon

**Keywords:** Routine health information system, Associated factors, Medical informatics, Medical informatics applications, Health informatics

## Abstract

**Background:**

Routine Health Information Systems (RHIS) of low-income countries function below the globally expected standard, characterised by the production and use of poor-quality data, or the non-use of good quality data for informed decision making. This has negatively influenced the health service delivery and uptake. This study focuses on identifying the factors associated with the performance of RHIS of the health facilities (HF) in Yaoundé, so as to guide targeted RHIS strengthening.

**Methods:**

A HF-based cross-sectional study in the 6 health districts (HDs) of Yaoundé was conducted. HFs were chosen using stratified sampling with probability proportional to size per HD. Data were collected, entered into Microsoft Excel 2013 and analysed with IBM- SPSS version 25. Consistency of the questionnaire was measured using Cronbach’s alpha coefficient. Pearson’s chi-square (and Fisher exact where relevant) tests were used to establish relationships between qualitative variables. Associations were further quantified using unadjusted Odd ratio (OR) for univariable analysis and adjusted odds ratio (aOR) for multivariable analysis with 95% confidence interval (CI). A *p*-value of less than 0.05 was considered statistically significant.

**Results:**

Of 111 selected HFs; 16 (14.4%) were public and 95 (85.6%) private. Respondents aged 24–60 years with an average of 38.3 ± 9.3 years; 58 (52.3%) males and 53(47.7%) females. Cronbach’s alpha was 0.96 (95%CI: 0.95–0.98, *p* < 0.001), proving that the questionnaire was reliable in measuring RHIS performances. At univariable level, the following factors were positively associated with good performances: *supportive supervision* (OR = 3.03 (1.1, 8.3); *p* = 0.02), *receiving feedback from hierarchy* (OR = 3.6 (0.99, 13.2); *p* = 0.05), *having received training on health information* (OR = 5.0 (1.6, 16.0); *p* = 0.003), and *presence of a performance evaluation plan* (OR = 3.3 (1.4, 8.2), *p* = 0.007). At multivariable level, the only significantly associated factor was *having received training on health information* (aOR = 3.3 (1.01, 11.1), *p* = 0.04).

**Conclusion:**

Training of health staff in the RHIS favors RHIS good performance. Hence, emphasis should be laid on training and empowering staff, frequent and regular RHIS supervision, and frequent and regular feedback, for an efficient RHIS strengthening in Yaoundé.

## Background

The health information system (HIS) makes up one of the pillars of the health system (HS) whose responsibility is the generation of data to facilitate the functioning of the other HS components: service delivery, health workforce, access to essential medicines, financing, and leadership [1]. A good HIS ensures the availability of good quality data and its use to support the informed decision-making process [2–4]. This highlights the importance of both the production of quality data and the use of the data for decision making at every level of the health pyramid. However, the RHIS of low-income countries experiences difficulties functioning at the globally expected standard, with respect to good data management and interpretation skills [5]. These systems are characterized by either the production and use of poor-quality data, or the non-use of good quality data by decision-makers, which negatively influence the delivery and uptake of health services [3–5]. Nevertheless, routine health information (RHI) availability permits a regular evaluation of the public health interventions both at the sub-national and national levels as well as an evaluation of HS strengthening interventions [6, 7] even though this has not been a regular practice in so many settings.

Since data generation is done at the level of the health facility, emphasis should be placed on the staff at this level through implicating them in the elaboration of RHI tools, planning, implementation, and evaluation of RHIS interventions. In Benin, exhaustibility of reporting increased from 16 to 89%, and the proportion of reports with sufficient data quality increased from 18.8 to 45.8% after implicating staff in the conception and elaboration of data collection tools [8]. However, RHIS staff englobes a wider range of personnel, including clinical, administrative, support staff as well as other users from different sectors whose roles cannot be overlooked [9].

State-related factors that would affect the functioning of an RHIS include governance, planning, availability of material, financial and human resources, supportive supervision, information dissemination, and promotion of a culture of information use [10]. The identification and definition of indicators, elaboration of tools for data collection, preparing procedural guides, and the mastery of hardware and software tool for data processing and analysis constitute the technical factors [10]. Behavioural determinants like HIS users’ demand, self-confidence, self-motivation, and competence to perform HIS tasks have also greatly influenced HIS performance and which require appropriate consideration and management accordingly [10–13]. Several African countries have sorted to harmonize and facilitate the HI collection through the putting into place of an open-source software platform. The most commonly solicited is the District Health Information System (DHIS) version 2, which has proven to improve the completeness of reporting [14].

The Cameroon health system is divided into 3 pyramidal levels. These include: the central (national), the intermediate (regional), and the peripheral (sub-regional) levels. Three sub-sections exist in the system: the public, private (private non-profit-making and private profit-making), and the traditional sub-sections [15, 16]. All these sub-sections are under the authority of the Ministry of Public Health. At each pyramidal level, there are administrative structures, health facilities, and also dialogue structures with linkage to the community [15]. The health facility is the point of entry of the clients (patient)s into contact with the health system and has as role to administer healthcare services to the clients.

The health sector is further segmented into 5 components (3 are vertical and 2 are horizontal). Vertical components are: health promotion, disease prevention, and disease management. Horizontal components include: health system strengthening, and governance [15]. Thus, services administered by the health facilities include those of the three vertical components of the health sector.

Following the standardization of data collection by the Cameroon Ministry of Public Health (MOPH) through the implementation of DHIS 2 [16], there is a tendency of improved HI management. This is due to the fact that both public and private HFs have as obligation to report through DHIS 2, thus improving on completeness and timeliness of reporting. Though not yet evaluated, this tool facilitates not just complete and timely reporting, but also facilitates feedback. Nevertheless, lack of management support, lack of skills by users, lack of computers, poor internet, and electricity coverage have been identified as potential challenges related to the efficient use of DHIS 2 [15].

Cameroon Health Sector Strategy (HSS) for the health information system (HIS) states: “*By 2027, ensure the development of health research and the availability of quality health information system for evidence-based decision-making at all levels of the health pyramid*” [16]. HSS aims at attaining 90% of health facilities having a well-organized system of data management [16]. To reduce the hindrances in meeting the HSS objectives and other health-related Sustainable Development Goals (SDGs) target for HIS, it is not only important to ensure the availability of RHI but also a performant RHIS that produces quality health information, as well as ensure its regular evaluation. Following Tamfon et al. [17] who determine the inadequate functionality of the RHIS of the HFs in Yaoundé, this study aims at identifying associated factors to good performance of the RHIS in Yaoundé; so as to guide targeted RHIS strengthening, and enable the re-orientation of the limited strengthening resources.

## Methods

### Study design and setting

We carried out a facility-based cross-sectional study for a period of 5 months extending from 1st May 2019 to 30th September 2019 in the 6 health districts (HDs) in Yaoundé. These HDs include: *Biyem-assi, Cité Verte, Djoungolo, Efoulan, Nkolbisson* and *Nkolndongo.*

### Study variables

The study variables were the socio-professional characteristics of participants, HF-related, and health system (HS)-related characteristics of participating HFs, and Health Facility and Community Information System Standards. Socio-professional characteristics included age, sex, professional qualification, years of experience, and function.

The independent variables were the HF-related, and health system (HS)-related characteristics: Status of the HF (public/private), Presence of health information unit ((HIU; (Yes/No)), Stable person in charge of statistics/data management (Yes/No), Available functional computer for data management (Yes/No), Stable internet (Yes/No), Availability of call credit (Yes/No), RHIS supervision received (Yes/No), Receiving feedback from hierarchy (Yes/No), Receiving training on HI (Yes/No), and Presence of a performance evaluation plan (PEP, Yes/No). A PEP is a written document that describes the process of carrying out the monitoring and evaluation of the RHIS performance, detailing the “What”, the “How”, and the “Why It Matters” for the RHIS evaluation, as well as exploiting evaluation results for RHIS performance improvement and decision making [18].

The dependent variable was the score of the Health Facility and Community Information System Standards. This variable was defined and classified into domains and subdomains by WHO and MEASURE Evaluation [19] as follows:

1. *Management and Governance (Policies and Planning, Management, Human Resources)*
2. *Data and Decision Support Needs (Data Needs, Data Standards)*
3. *Data Collection and Processing (Data Collection and Management of Individual Client Data; Collection, Management and Reporting of Aggregated Facility Data; Data quality assurance; Information and Communication Technology (ICT))*
4. *Data Analysis, Dissemination, and Use (Analysis, Dissemination, Data Demand and Use)*

The score of all the domains was calculated as follows [20]:

i) Proportion of scores for the various subdomains: we summed the score for each item (question) in the subdomain, multiplied by 100 and divided by the maximum score for that subdomain.
ii) Proportion of scores for the domains: we summed up the obtained scores of the subdomains in the given domain, multiplied by 100 and divided by the maximum score for that domain.
iii) Global score for all the domains, we summed up all the obtained scores of all the domains, multiplied by 100 and divided by the maximum global score. The obtained global score was then grouped into good score (scoring 60% and above) and poor score (less than 60%).

### Sample size and sampling

A minimum sample size (n) of 106 HF that were visited was obtained using the formula: $n=\frac{Z^2\times P\left(1-P\right)}{d^2}$ [21], where Z is the quantile of the normal distribution at 5% level which equals to 1.96, P is the proportion of adequately functioning HFs which is 10% [22], d is the precision = 0.06 [21], and non-response rate of 10%. HFs were selected through a stratified sampling that uses probability proportional to size in each HD. The two stratified variables were HD and HF status (Private, Public).

The recruitment criteria for HFs included functional public and private HFs of the operational level whose consent for participation was obtained. Respondents (one per HF) were either the head, person in charge of statistics/data management, or any other responsible staff, capable to provide the needed responses.

### Data collection

Interviewers were recruited and trained to understand the objectives and the methodology of the study. Data were collected using the WHO/MEASURE Evaluation pre-established Rapid Assessment questionnaire [19] that was slightly modified to include the socio-professional characteristics of respondents, HF and HS-related characteristics. Each question was scored as: *0 (no answer/not applicable); 1 (not present, needs to be developed); 2 (needs a lot of strengthening); 3 (needs some strengthening); and 4 (already present, no action needed).*

### Statistical data analysis

Data were entered into Microsoft Excel 2013, cleaned and then exported for analyses using IBM-SPSS version 25 [23]. Frequencies and percentages (%) were used to describe qualitative variables. Consistency of the RHIS assessment tool in measuring the gaps and weaknesses in the RHIS was measured using the Cronbach’s alpha (α) whose score is comprised between 0 and 1, and was interpreted as [24, 25]: (i) *unacceptable* if α < 0.7, (ii) *acceptable* if 0.7 ≤ α < 0.8, (iii) *good* if 0.8 ≤ α < 0.9, (vi) *excellent* if α ≥ 0.9. Pearson’s Chi-square test (Fisher exact test where relevant) was used to establish relationships between qualitative variables. Associations were further quantified using unadjusted Odds ratio (OR) for univariable analysis and adjusted Odds ratio (aOR) for multiple logistic regression analysis with 95% confidence interval (CI). The Hosmer-Lemeshow (HL test)‘s goodness of fit test was used to assess the adequacy of the multiple logistic regression. A *p*-value of less than 0.05 was considered statistically significant.

### Ethical considerations

The study received ethical approval CE N^0^ 00786/CRERSHC/2019 from the Regional Research Ethics Committee for Human Health of the Centre and the authorization N^0^ 00756−/AP/MINSANTE/SG/DRSPC/CRERSH from the Regional Delegate of Public Health for the Centre Region. Study procedures were described to participants, during which they were briefly and clearly informed of their voluntary participation in the study; and that refusal to participate would have no negative consequences. Their informed consents were then obtained prior to the interview.

## Results

Of 111 selected HFs (out of 799); 16 (14.4%) were public and 95 (85.6%) were private. Respondents aged 24–60 years with an average of 38.3 ± 9.3 years; 58 (52.3%) males and 53(47.7%) females.

### Distribution of health system-related characteristics

As presented in Table 1, *HIU*, *stable internet* and *PEP* were available only in 27.9, 39.6 and 27.9% of HFs respectively. The other characteristics: *having received training on HI* (63.1%), *feedback received from hierarchy* (74%), *RHIS supervision* (60.4%), and *presence of functional computer* (55.9%) were present in more than 50% but less than 75% of the HFs. However, stable persons in charge of statistics/data management were available in 78.4% of HFs.

**Table 1:** Availability of health system-related characteristics

Health system-related characteristics	Proportions (%)	
	Yes	No
PEP available	27.9	72.1
Trained on HI	63.1	36.9
Feedback received	74.8	25.2
RHIS Supervision	60.4	39.6
Call credit available	62.2	37.8
Stable internet	39.6	60.4
Functional Computer	55.9	44.1
Stable data manager	78.4	21.6
HIU present	27.9	72.1

*PEP* Performance Evaluation Plan, *HI* Health information, *RHIS* Routine health information system, *HIU* Health information unit

### Cronbach’s analysis

The consistency of the items of the RHIS tool was assessed using Cronbach’s alpha. Overall Cronbach’s alpha was 0.96 (95%CI: 0.95–0.98, *p* < 0.001), proving that the questionnaire was reliable in measuring RHIS performances. Table 2 shows Cronbach’s alpha for various domains and subdomains; they vary for subdomains between 0.68 (*Collection and Management of Individual Client Data*) and 0.88 (*Management*); and for domains between 0.90 (*Data Analysis, Dissemination, and Use*) and 0.93 (*Management and Governance*).

**Table 2:** Presentation of consistency of items using Cronbach’s alpha

	Domains and subdomains	Cronbach’s alpha (95% CI) **
**1**	**Management and Governance**	0.93 (0.89–0.95)
1.1	Policies and Planning	0.87 (0.81–0.92)
1.2	Management	0.88 (0.85–0.91)
1.3	Human Resources	0.83 (0.78–0.88)
**2**	**Data and Decision Support Needs**	0.91 (0.88–0.93)
2.1	Data Needs	0.88 (0.84–0.91)
2.2	Standards and System Design	0.83 (0.77–0.87)
**3**	**Data Collection and Processing**	0.91 (0.87–0.93**)**
3.1	Collection and Management of Individual Client Data	0.68 (0.58–0.77)
3.2	Collection, Management, and Reporting of Aggregated Facility Data	0.81 (0.74–0.86)
3.3	Data Quality Assurance	0.87 (0.83–0.90)
3.4	Information and Communication Technology (ICT)	0.71 (0.62–0.79)
**4**	**Data Analysis, Dissemination, and Use**	0.90 (0.87–0.93)
4.1	Data Analysis	0.82 (0.76–0.87)
4.2	Information Dissemination	0.79 (0.72–0.84)
4.3	Data Demand and Use	0.76 (0.68–0.82)

**All *p*-values were less than 0.001

### Factors associated with RHIS performance

Table 3 shows that private non-profit-making HFs show a better performance (37.5%) compared to public (26.7%) and private profit-making (25.0%) HFs (*p* = 0.52). HFs with a HIU performed slightly poorer (22.6%) compared to HFs without a HIU (28.8%), with non-significant difference (*p* = 0.50). Facilities with stable persons in charge of statistics (27.7%), functional computer (31.7%), stable internet (35.7%), and call credit (30.3%) performed better compared to those that did not possess the resources or benefitted from activities (*p* = 0.56, *p* = 0.30, *p* = 0.80, and *p* = 0.35 respectively). Facilities that benefitted from RHIS supervision (34.8%), received feedback from hierarchy (32.1%), facilities trained on HI (36.8%) and those with a PEP (45.2%) also presented better and statistically significant performances with respect to HFs that did not. Thus, the following factors were positively associated with good performances after univariable analysis: *RHIS supervision* (OR = 3.03 (1.1, 8.3); *p* = 0.02), *receiving feedback from hierarchy* (OR = 3.6 (0.99, 13.2); *p* = 0.05), *having received training on health information* (OR = 5.0 (1.6, 16.0); *p* = 0.003), and *presence of a performance evaluation plan* (OR = 3.3 (1.4, 8.2), *p* = 0.007). At multivariable level, the only significantly associated factor was *having received training on health information* (aOR = 3.3 (1.01, 11.1), *p* = 0.04). The Hosmer-Lemeshow’s goodness of fit test showed that the multiple logistic model adequately fitted the data (Chi-squared test = 5.02, df = 5, *p* = 0.4).

**Table 3:** Associated factors to the RHIS performance after univariable and multivariable analysis

Variables	Good	Poor	Total	Univariable		Multivariable	
				OR (95%CI)	P	aOR (95%CI)	P
**Status**							
*Public*	4 (26.7)	11 (73.3)	15 (100.0)	1			
*Private NPM*	6 (37.5)	10 (62.5)	16 (100.0)	0.6 (0.1, 2.8)	0.52		
*Private PM*	19 (25.0)	57 (75.0)	76 (100.0)	1.09 (0.3, 3.8)	0.89		
**HIU present**							
*Yes*	7(22.6)	24(77.4)	31(100)	0.7(0.3,1.9)	0.50		
*No*	22(28.8)	54(71.2)	76(100)	1			
**Stable person in charge of statistics**							
*Yes*	23 (27.7)	60 (72.3)	83 (100)	1.4 (0.46,4.2)	0.56		
*No*	5 (21.7)	18 (78.3)	23 (100)	1			
**Functional computer**							
*Yes*	19 (31.7)	41 (68.3)	60 (100)	1.76 (0.7, 4.2)	0.30		
*No*	10 (21.3)	37 (78.7)	47 (100)	1			
**Stable Internet**							
*Yes*	15 (35.7)	27 (64.3)	42 (100)	2.2 (0.9, 5.2)	0.80		
*No*	13 (20.3)	51 (79.7)	64 (100)	1			
**Call credit**							
*Yes*	20 (30.3)	46 (69.7)	66 (100)	1.5 (0.6, 3.9)	0.35		
*No*	9 (22.0)	32 (78.0)	41 (100)	1			
**RHIS Supervision**							
*Yes*	23 (34.8)	43 (65.2)	66 (100)	3.03 (1.1, 8.3)	0.02	1.9 (0.6, 5.6)	0.2
*No*	6 (15.0)	34 (85.0)	40 (100)	1		1	
**Feedback received**							
*Yes*	26 (32.1)	55 (67.9)	81 (100)	3.6 (0.99, 13.2)	0.05	2.0 (0.5, 8.0)	0.3
*No*	3 (11.5)	23 (88.5)	26 (100)	1		1	
**Trained on HI**							
*Yes*	25 (36.8)	43 (63.2)	68 (100)	5.0 (1.6, 16.0)	0.003	3.3 (1.01,11.1)	0.04
*No*	4 (10.3)	35 (89.7)	39 (100)	1		1	
**PEP**							
*Yes*	14 (45.2)	17 (54.8)	31(100)	3.3 (1.4, 8.2)	0.007	2.3 (0.9, 6.0)	0.09
*No*	15 (19.7) 1 1	61 (80.3)	76(100)	1		1	

## Discussion

This study assessed the factors associated with the performance of the RHIS as a whole component of the health system. It was evidenced that HFs that have benefited from HI training were more likely to perform better than their counterparts. Training on HI enables staff to acquire skills in basic computer use, planning, data analysis and management, data use, and interpretation. This also includes training on DHIS 2 which is the software used by the Ministry of Public Health for health data management. In a cross-sectional descriptive study carried out in Kenya [14], 80.8% of the participants agreed or strongly agreed that the use of DHIS 2 by already trained workers improved the performance of the staff. Similar findings were obtained in Northwest Ethiopia [26] and Western Ethiopia [27]. Taking into consideration the increased need to train staff and the limited financial resources for this process, Tamfon et al. [17] suggested less costly training approaches which include online courses and video-recorded sessions that can be used for self-training, and also training of the district officer and giving them the responsibility to train their staff on regular bases during HD coordination meetings.

Sixty percent of HFs reported having been supervised, even though most of these facilities (34.8%) presented better performance compared to their counterparts. This calls to question the quality and frequency of the supervision visits. Supervision aims to empower and support staff in carrying out their daily tasks [28]. This should be done in a regular manner with a checklist that includes all the aspects of the system. It was noticed that supervision irregularities were responsible for the poor performance of HMIS in Ethiopia [28]. This phenomenon applied to sending feedback by the hierarchy on the quality of forwarded data and the findings post supervision visit. Our study found that nearly a quarter of HFs acknowledged having received feedback from the superiors. This finding is similar to that of a study in Kenya, where 72% of the respondents stated to have received feedback concerning their reports from the superiors at unscheduled [29]. Also, Cheburet and Odhiambo-Otieno [29] obtained a statistically significant association between the feedback of data and frequency of feedback (*p* < 0.001). We noticed that 32.1% of HFs who have received feedback from superiors presented a good performance compared to 11.5% among HFs that did not receive feedback. It however points out the importance of regular and frequent feedback mechanisms to enable immediate discussion and resolution of problems for RHIS strengthening [30].

Functional computers for data management was present in about half (55.9%) of the health facilities. Of these facilities, 31.7% had a good performance against 21.3% among facilities without one. This finding was similar to that obtained by Shiferaw et al. [26] in Ethiopia where he evidenced that the presence of a computer in a health department was associated with good use of health information [26]. However, the availability of a functional computer was proven to be significantly associated with HI use. In this study by Asemahgn [24] in Western Amhara of Ethiopia, facilities that had a computer were more likely to use HI for decision making, compared to those that did not have a computer. This difference in the findings could be explained in part by the differences in the outcome variable under investigation and also by the differences in the tools used. As earlier stated, we explored the performance of the RHIS domains as a whole, meanwhile, Asemahgn focused only on HI use. Nevertheless, both findings stress the importance of having a computer for data management in the HFs and departments. Lack of computers, as well as other factors, were also some challenges in strengthening the district-based health reporting through DHIS 2 in Uganda [31].

The presence of a functional computer alone in an HF or department will not guarantee effective and efficient HI management. This is complemented by the services of a stable and computer literate person in charge of statistics. The difficulties experienced by most HFs are that persons in charge of data management are unstable, untrained in HI management and computer skills, or occupy other posts [28]. Apart from this problem, our study indicates that HFs with stable persons in charge of statistics were more likely to perform better than their counterparts.

DHIS 2 was implemented with the hope of meeting the health sector strategic objective of attaining 80% completeness in monthly activities reporting by 2027 [16]. With this platform, only an internet connection can permit reports forwarding. There is good coverage in Yaoundé, but HFs located in remote areas have poor internet coverage. Our findings show that HFs with stable internet were likely to perform better than their counterparts. However, the problem of HFs in Yaoundé is not poor coverage, but rather the availability of internet communication cost. Though limited in resources, HD officers have been provided telephones with communication credit for communication with HFs. This improved performance with respect to HFs reporting and feedback sending by hierarchy.

Some limitations of the study include the fact that it didn’t focus enough on other behavioural determinants such as the knowledge, skills, attitudes, values, and motivation of the people who collect, analyse, and use health data. In addition, our cross-sectional study design prevented the findings from showing causal relationships.

## Conclusion

This study revealed that training of health staff in the RHIS is a determinant of the good performance of the RHIS system in Yaoundé. In this light, emphasis should be laid on training and empowering staff. Likewise, frequent and regular RHIS supervision, and frequent and regular feedback should also be implemented for an efficient RHIS strengthening in Yaoundé. Health authorities should carry the greater responsibility in the strengthening process through the creation of an enabling environment. This involves advocating and providing sufficient resources, monitoring, and evaluation of the implementation of RHIS policies by both public and private HFs for better management of data for evidence-based decisions making. However, the findings of this study also pave the way for further research in the domain of RHIS. Post-implementation evaluation studies will be better indicated to measure the change in the performance of the RHIS after the implementing these interventions. Secondly, cost-effective studies will be indicated to measure the cost effectiveness of these interventions especially in a low-income country setting.

## Data Availability

The datasets used and/or analyzed during the current study are available on reasonable request.

## Abbreviations

CI: Confidence Interval
DHIS2: District Health Information System 2
HD: Health District
HF: Health Facility
HI: Health Information
HS: Health System
HIS: Health Information System
HIU: Health Information Unit
HMIS: Health Management Information System
HSS: Health Sector Strategy
ICT: Information Communication Technology
MOPH: Ministry of Public Health
OR: Odds Ratio
PEP: Performance Evaluation Plan
RHI: Routine Health Information
RHIS: Routine Health Information System
SDG: Sustainable Development Goals
WHO: World Health Organisation
